# Supplementary material for: Solution-processed intermediate-band solar cells with lead sulfide quantum dots and lead halide perovskites
Source: Nat Commun. 2019 Jan 10;10:43. doi: 10.1038/s41467-018-07655-3 (PMC6327045; doi:10.1038/s41467-018-07655-3)
Supplement: Supplementary file 1 — Supplementary Information [file 41467_2018_7655_MOESM1_ESM.pdf]

Supplementary Information for

Solution-processed intermediate-band solar cells with lead sulfide quantum dots and lead halide perovskites

Hiroji Hosokawa*, Ryo Tamaki, Takuya Sawada, Akinori Okonogi, Haruyuki Sato, Yuhei Ogomi, Shuzi Hayase, Yoshitaka Okada, Toshihiro Yano

correspondence to: hosokawa.hiroji@kao.com (H. H.)

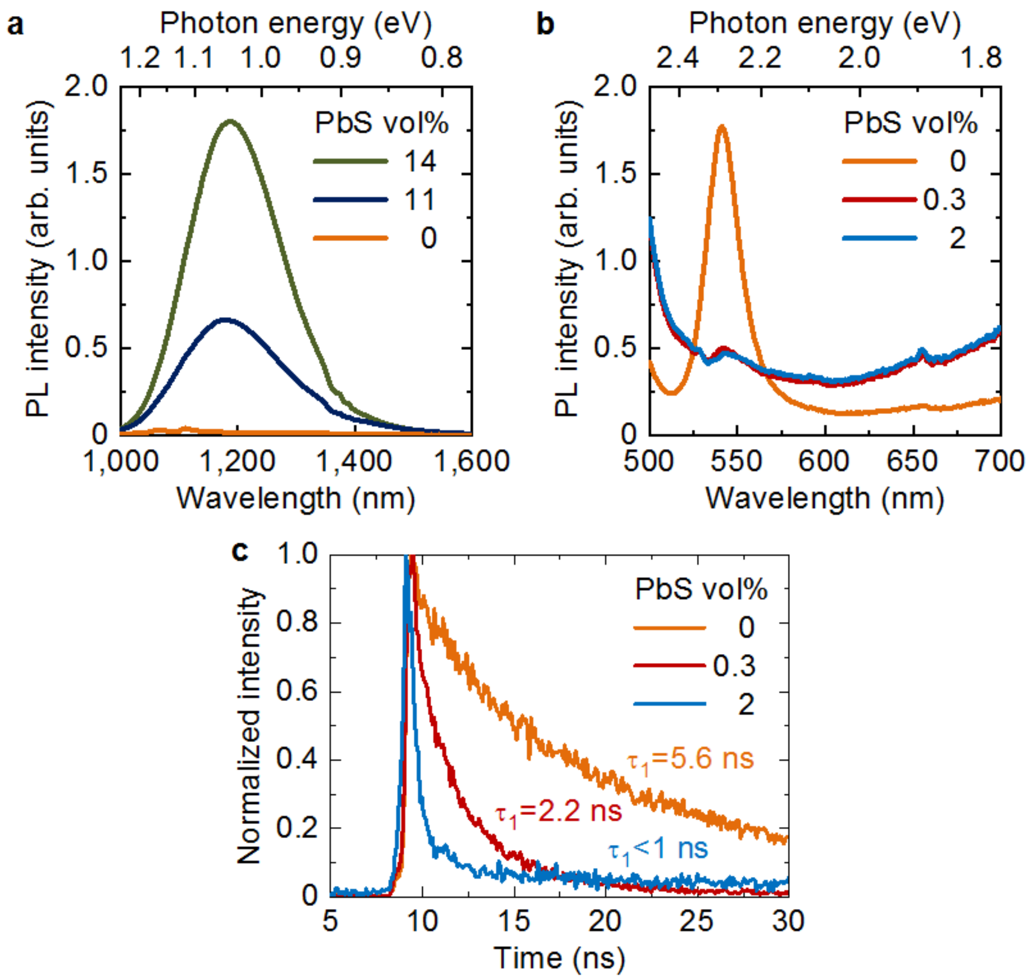

Supplementary Figure 1 PL properties of the photo-absorption layers at room temperature. **a** NIR PL spectra with the excitation wavelength of 532 nm. **b** Visible light PL spectra with the excitation wavelength of 480 nm. **c** Time-resolved PL decay curves with the excitation wavelength of 470 nm, the monitor wavelength of 544 nm, and the time range from 0 to 100 ns.

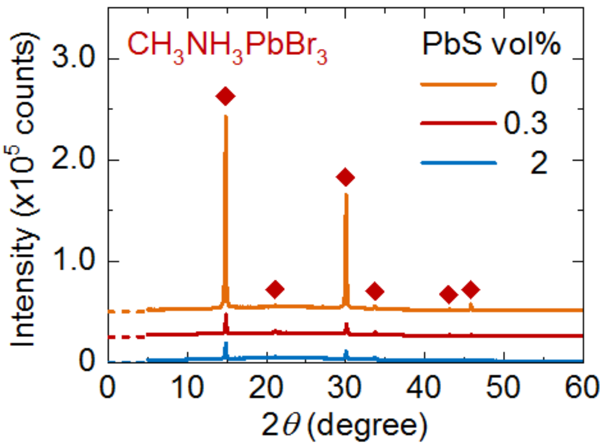

Supplementary Figure 2 XRD patterns of the photo-absorption layers on glass substrates. The peaks at $2\theta = 14.8, 21.1, 30.0, 33.7, 43.0, 45.8$ degree correspond to the (100), (110), (200), (210), (220), (300) reflections of cubic $CH_3NH_3PbBr_3$ perovskite.

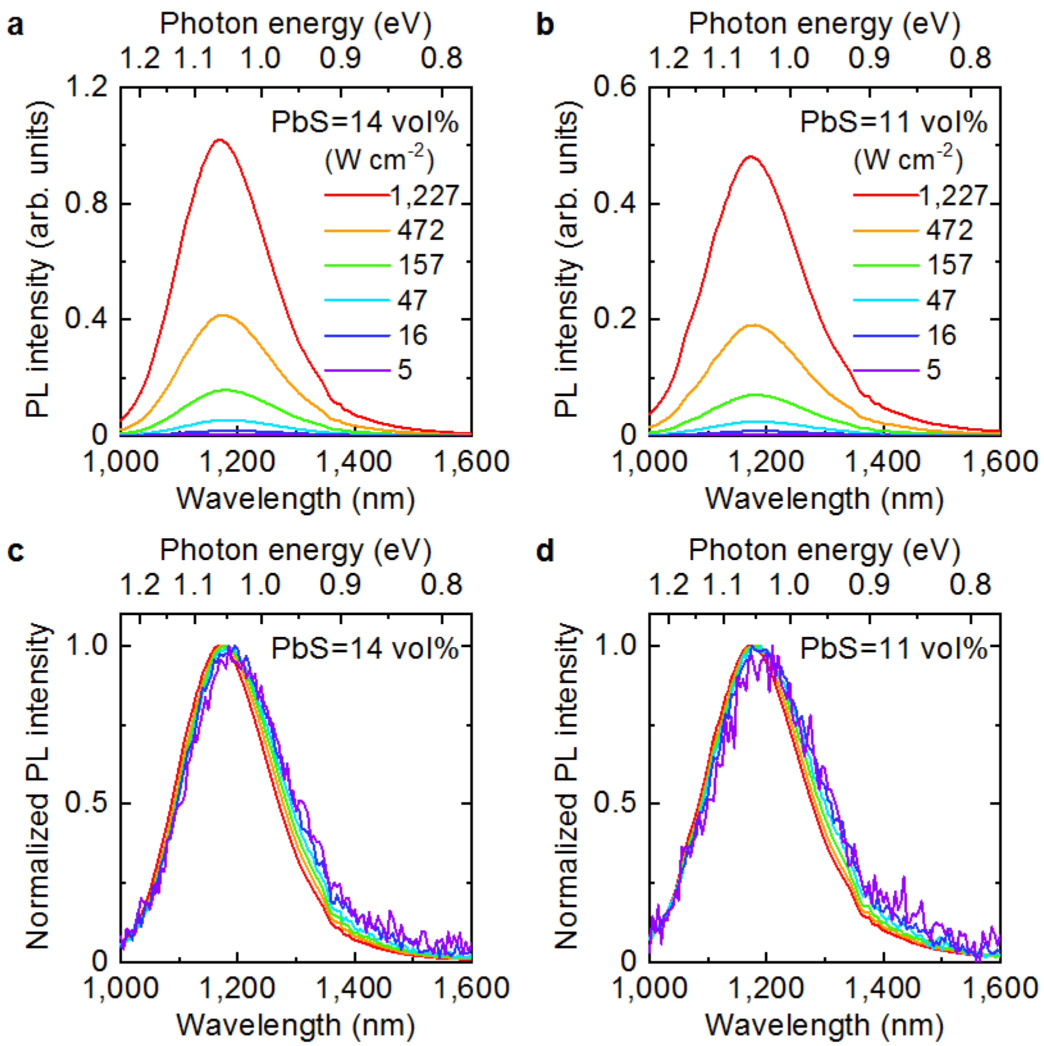

Supplementary Figure 3 Excitation density dependence of NIR PL spectra of the photo-absorption layers at room temperature. The PbS volume concentrations in the photo-absorption layers were 14 volume% (**a**, **c**) and 11 volume% (**b**, **d**), respectively. Normalized PL spectra are also shown in (**c**) and (**d**). Excitation wavelength was 532 nm.

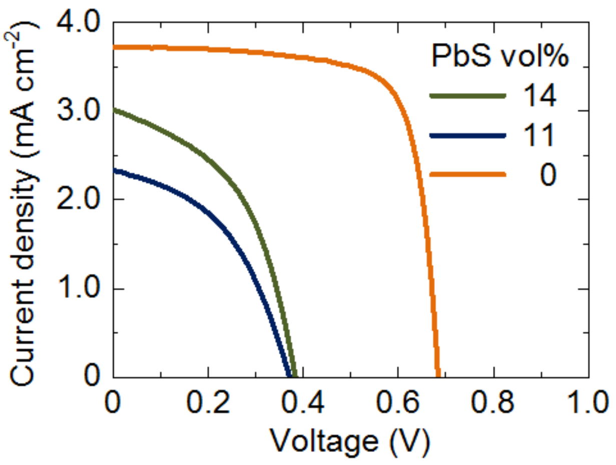

PbS (vol%)	14	11	0
PCE (%)	0.55	0.39	1.9
V_{OC} (V)	0.38	0.37	0.68
J_{SC} (mA cm^{-2})	3.0	2.3	3.7
FF	0.48	0.45	0.75

Supplementary Figure 4 Photocurrent density-voltage (J-V) curves and photovoltaic parameters of solution-processed IBSCs with PbS QDs and $CH_3NH_3PbBr_3$ perovskite. The J-V curves were recorded at a scanning rate of 0.1 V s^{-1} in forward direction under standard AM 1.5G illumination.

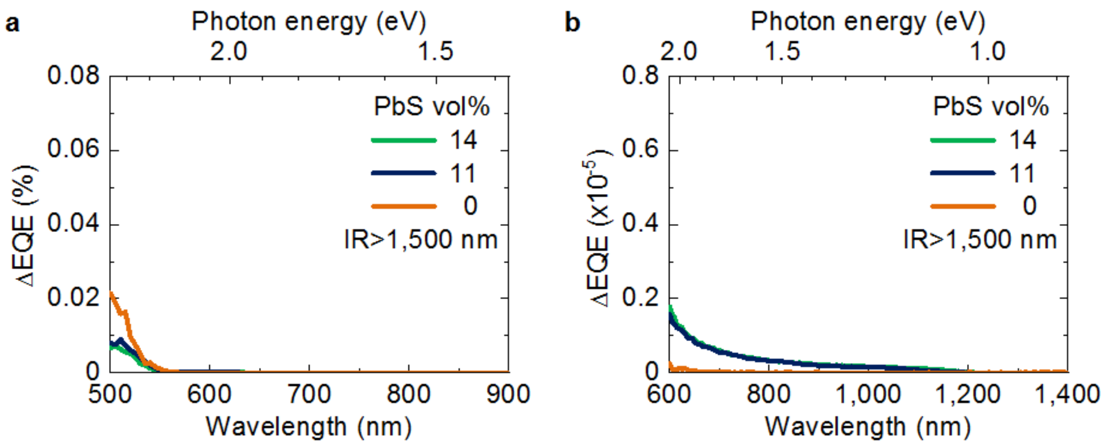

Supplementary Figure 5 Room-temperature ΔEQE spectra by using IR bias light with more than 1,500 nm. **a** ΔEQE spectra from 500 to 900 nm. **b** Magnified ΔEQE spectra from 600 to 1,400 nm.

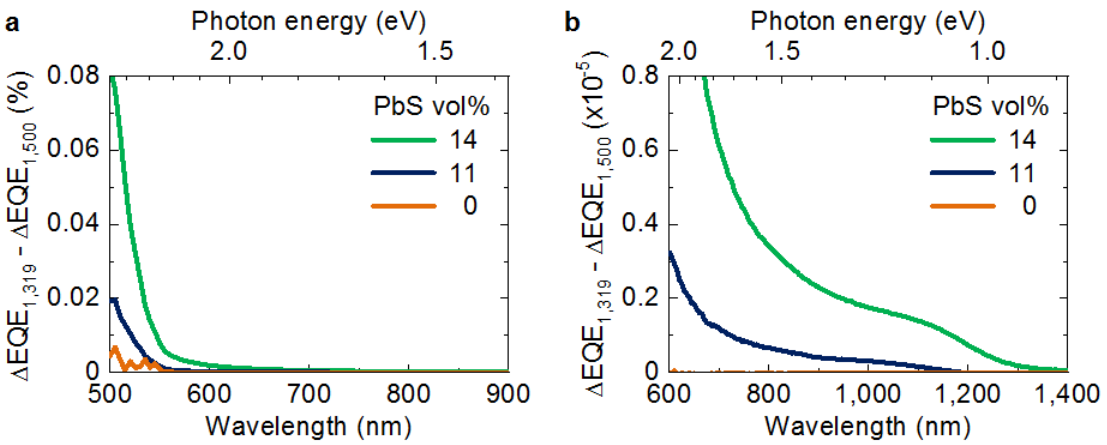

Supplementary Figure 6 Difference spectra in ΔEQE by using IR bias light between more than 1,319 nm and more than 1,500 nm. **a** Difference spectra from 500 to 900 nm. **b** Magnified difference spectra from 600 to 1,400 nm.
